# The Pivotal Role of Plant Derivatives and Eicosanoid Signaling Modulation in Counteracting Cardiomiopathy

**DOI:** 10.3390/ijms27114849

**Published:** 2026-05-28

**Authors:** Sara Ussia, Giovanna Ritorto, Roberta Macrì, Maria Serra, Annamaria Tavernese, Carmen Altomare, Denise Maria Dardano, Chiara Idone, Ernesto Palma, Carolina Muscoli, Maurizio Volterrani, Francesco Barillà, Vincenzo Mollace, Rocco Mollace

**Affiliations:** 1 Department of Health Sciences, Institute of Research for Food Safety and Health (IRC-FSH), University “Magna Graecia” of Catanzaro, 88100 Catanzaro, Italycarmen.altomare@studenti.unicz.it (C.A.); denisemaria.dardano@studenti.unicz.it (D.M.D.);; 2 Laboratory of Mass Spectrometry and Proteomics, Department of Health Sciences, University “Magna Græcia” of Catanzaro, 88100 Catanzaro, Italy; 3 Department of Medicine and Surgery, University Campus Bio-Medico of Rome, 00128 Rome, Italy; 4 Veterinary Pharmacology Laboratory, Department of Health Sciences, Institute of Research for Food Safety and Health (IRC-FSH), University “Magna Graecia” of Catanzaro, 88100 Catanzaro, Italy; 5 Cardiology Department, IRCCS San Raffaele Roma, 00166 Rome, Italy; maurizio.volterrani@sanraffaele.it; 6 Department of Experimental Medicine, University “Tor Vergata” of Rome, 00133 Rome, Italy; 7 Renato Dulbecco Institute, 88046 Lamezia Terme, Italy

**Keywords:** eicosanoids, eicosanoid receptors, cardiomyopathy, cardiomyocytes, nutraceutical supplementation, natural derivatives

## Abstract

Eicosanoids and their receptors act as key regulators of inflammation, calcium homeostasis, mitochondrial function, and cardiomyocyte survival, thereby contributing to the onset and progression of cardiac dysfunction. This review aims to summarize the evidence to underscore the pivotal role of eicosanoids and their receptors in the pathophysiology of cardiomyopathy, analysing the potential protective activity of traditional and natural compounds to counteract cardiovascular disease onset and progression. Among eicosanoid receptors, prostaglandin E2 receptor 3 (EP3), prostaglandin E2 receptor 4 (EP4), chemoattractant receptor expressed on type 2 helper T cells (CRTH2), and thromboxane prostanoid (TP) emerge as critical modulators with distinct and often opposing effects on cardiac physiology. While EP3 and CRTH2 are predominantly associated with detrimental outcomes such as impaired contractility and enhanced apoptosis, EP4 signalling consistently demonstrates cardioprotective properties, including improved calcium handling and preservation of mitochondrial integrity. These findings highlight the therapeutic potential of selectively targeting eicosanoid receptor pathways to mitigate cardiac remodelling and dysfunction. In parallel, increasing attention has been directed toward natural bioactive compounds as complementary strategies for cardioprotection. Polyphenols, flavonoids, carotenoids, and other nutraceuticals exert beneficial effects through antioxidant, anti-inflammatory, and anti-apoptotic mechanisms, often intersecting with eicosanoid signalling pathways. Their ability to modulate oxidative stress and inflammatory responses suggests a promising role in preventing or attenuating cardiomyopathy, particularly in metabolic and drug-induced contexts. Future research should focus on well-designed clinical trials, a deeper characterization of receptor-specific signalling networks, and the development of targeted therapies that combine pharmacological and nutraceutical approaches. Overall, a better understanding of eicosanoid-mediated mechanisms may open new ways for cardiomyopathy prevention and treatment, ultimately improving patient outcomes and reducing the burden of cardiovascular disease.

## 1. Introduction

Cardiomyopathy is defined as ‘a myocardial disorder in which the heart muscle is structurally and functionally abnormal, in the absence of coronary artery disease (CAD), hypertension, valvular disease, and congenital heart disease (CHD) sufficient to cause the observed myocardial abnormality’ [1]. In 2006, the American Heart Association (AHA) defined cardiomyopathies as a heterogeneous group of myocardial diseases associated with mechanical and/or electrical dysfunction, which usually (though not invariably) present with inappropriate ventricular hypertrophy or dilatation. It is important to note that cardiomyopathies can coexist with ischaemic, valvular, and hypertensive disease and that the presence of one does not exclude the possibility of the other [2]. Myocardial dysfunction is the major detrimental factor in heart disease, often resulting in Heart Failure (HF) and a higher risk of sudden cardiac death (SCD), which represents a significant cause of death in cardiomyopathies, even with improved survival rates due to current treatments [3]. A more accurate diagnosis can be made by assessing the clinical scenario and the morphological and functional characteristics of the ventricle, as well as analysing additional elements such as arrhythmias, conduction disorders, family history, genetic testing, extracardiac involvement, and laboratory markers [4]. The World Health Organization (WHO) classifies cardiomyopathies according to their morphology, functional abnormalities, and the underlying genetic or acquired aetiologies, into primary and secondary cardiomyopathies [5]. Primary cardiomyopathies include conditions in which the heart is the main organ affected, often on a genetic or idiopathic basis, and include dilated cardiomyopathy (DCM), characterised by ventricular dilation and reduced ejection fraction (EF), hypertrophic cardiomyopathy (HCM), with wall thickening and diastolic dysfunction, restrictive cardiomyopathy (RCM), marked by myocardial stiffness and impaired ventricular filling, arrhythmogenic right ventricular cardiomyopathy (ARVC), in which muscle tissue is replaced by fibroadipose tissue, predisposing to severe arrhythmias and non-dilated ventricular cardiomyopathy (NDLVC), in which ventricular dysfunction occurs without significant dilation [6]. Secondary cardiomyopathies, on the other hand, result from extracardiac conditions that secondarily damage the myocardium, such as metabolic diseases (e.g., diabetes mellitus (DM), amyloidosis), endocrinopathies (hyperthyroidism, pheochromocytoma), autoimmune and inflammatory diseases (sarcoidosis), drug or substance toxicity (chemotherapy, alcohol), and infections (viral myocarditis) [6]. This distinction has significant implications for differential diagnosis, clinical management, and prognosis. Therapeutic strategies for primary cardiomyopathies focus on mitigating cardiac dysfunction and reducing the risk of arrhythmias and HF. In addition, effective care for secondary cardiomyopathies requires addressing the underlying systemic condition alongside providing targeted cardiac support. It has been demonstrated that eicosanoids are versatile bioactive lipid mediators that participate in numerous physiological processes, contributing to the regulation of renal, cardiovascular, and gastrointestinal functions and to the modulation of vascular tone, blood flow, blood pressure, and pain perception [7]. Based on these findings, this review aims to provide a comprehensive overview of current evidence on the role of eicosanoids and their receptors in the pathophysiology of cardiomyopathies. In particular, this study mapped and synthesised existing literature on the molecular and cellular mechanisms through which eicosanoids influence cardiac metabolism, mitochondrial function, and myocardial remodelling. Furthermore, critical analysis of prostaglandin E2 receptor 3 (EP3) and prostaglandin E2 receptor 4 (EP4) receptor involvement in the progression of cardiomyopathies was carried out with emphasis on their dual effects on contractility, inflammation, and fibrosis. Finally, this review identified clinical implications and future perspectives, evaluating the potential of eicosanoids as diagnostic biomarkers and therapeutic targets.

### 1.1. Eicosanoids and Chemical Characterization

Eicosanoids are oxidation products derived from C20 polyunsaturated fatty acids (PUFAs), primarily arachidonic acid (AA), and, in specific contexts, docosahexaenoic acid (DHA). These lipid mediators constitute a remarkably diverse and biologically potent family of signalling molecules, exerting rapid and context-dependent actions across virtually all tissues [8]. Eicosanoids participate in the regulation of several processes involved in cardiac physiology and myocardial homeostasis, including vascular tone, platelet function, cardiomyocyte contractility, calcium handling, mitochondrial activity, immune cell communication, inflammatory signalling, and cellular responses to oxidative stress [9]. Through the modulation of arachidonic acid-derived pathways, eicosanoids contribute to the control of myocardial adaptation and repair mechanisms; however, their dysregulation may promote pathological hypertrophy, myocardial fibrosis, maladaptive ventricular remodelling, and progressive cardiac dysfunction. Increasing evidence also suggests that altered eicosanoid signalling plays an important role in the onset and progression of cardiomyopathy and heart failure by influencing inflammatory cascades, extracellular matrix deposition, and cardiomyocyte survival [9]. Their ability to both trigger and resolve inflammatory responses highlights their dual role in maintaining tissue homeostasis. Although short-lived, these molecules act as highly efficient intra- and intercellular signals, and even their downstream metabolites can function as secondary messengers [10]. The generation of eicosanoids is initiated by the release of PUFA precursors from membrane phospholipids through enzymatic hydrolysis mediated by phospholipases. Among these enzymes, phospholipases A_2_ (PLA_2_s) represent the principal catalysts responsible for the selective cleavage of the sn-2 ester bond of glycerophospholipids, a position typically enriched in PUFA [11]. PLA_2_s comprise multiple isoforms, including cytosolic phospholipase A_2_ (cPLA_2_), secreted phospholipases A_2_ (sPLA_2_), and calcium-independent phospholipases A_2_ (iPLA_2_), each characterized by distinct regulatory mechanisms, substrate specificities, and subcellular distributions [12]. Their activation is regulated by intracellular Ca^2+^ fluctuations, phosphorylation events, and stimulus-dependent membrane translocation, ensuring that PUFA mobilization occurs with spatial and temporal precision. Through regulated hydrolysis, PLA_2_s provide AA and other PUFAs for oxylipin synthesis while also contributing to membrane remodelling and the formation of lysophospholipid mediators, thereby influencing both structural and signalling properties of the cell [13]. Once released, AA and other PUFAs serve as substrates for the biosynthesis of several major classes of bioactive lipid mediators, including prostaglandins (PG), thromboxanes (TX), leukotrienes (LT), hydroperoxy fatty acids (HpFA), lipoxins, epoxyeicosatrienoic acids (EETs), and hydroxyeicosatetraenoic acids (HETEs). The enzymatic conversion of PUFA into eicosanoids occurs through highly stereo- and regio-specific oxidation reactions catalyzed by three major enzymatic families: cyclooxygenases (COX), lipoxygenases (LOX), and cytochrome P450 monooxygenases (CYP450) [14,15]. Among oxylipins, eicosanoids represent a group with remarkable structural diversity and functional significance. Their molecular architecture is characterized by highly unsaturated hydrocarbon chains whose double bond position and geometry strongly influence enzyme recognition and product specificity [16]. Consequently, even subtle structural variations may result in distinct biological effects, highlighting the close relationship between chemical structure and functional activity. The COX pathway is one of the most extensively studied routes involved in eicosanoid biosynthesis. COX enzymes convert AA into the unstable intermediate prostaglandin G_2_ (PGG_2_), which is rapidly reduced to prostaglandin H_2_ (PGH_2_), the common precursor of several prostanoids, including prostaglandin D_2_ (PGD_2_), prostaglandin E_2_ (PGE_2_), prostaglandin F2α (PGF_2_α), prostaglandin I2 (PGI_2_), and thromboxane A2 (TXA_2_)/thromboxane B2 (TXB_2_) [17]. Among the two main isoforms, COX-2 is strongly induced under inflammatory and pathological conditions [18]. In parallel, the LOX pathway contributes to eicosanoid diversification through the activity of 5-, 12-, and 15-LOX isoforms, which catalyze the oxygenation of AA to generate hydroperoxyeicosatetraenoic acids (HPETEs). These intermediates serve as precursors for leukotrienes, including leukotriene A4 (LTA_4_), leukotriene B4 (LTB_4_), leukotriene C4 (LTC_4_), leukotriene D4 (LTD_4_), and leukotriene E4 (LTE_4_), mainly involved in inflammatory and immune responses. The same pathway also generates lipoxins, such as lipoxin A_4_ (LXA_4_) and lipoxin B₄ (LXB_4_), molecules with anti-inflammatory and pro-resolving properties that counterbalance the actions of pro-inflammatory eicosanoids [19]. The CYP450 pathway further expands the diversity of eicosanoid mediators through the formation of EETs and HETEs via epoxygenation and hydroxylation reactions of AA. Although less extensively investigated than PG and LT, these metabolites are increasingly recognized for their involvement in inflammation, oxidative stress regulation, and tissue homeostasis [15]. Collectively, the COX, LOX, and CYP450 pathways generate a highly integrated and dynamic network of lipid mediators whose production depends on cellular context, enzyme specificity, tissue distribution, and precursor availability [9,20]. The AA cascade and local tissue environment ultimately determine the specific eicosanoid profile within each cell, allowing rapid adaptation to physiological and pathological stimuli.

### 1.2. Eicosanoid Receptors

**Figure 1:**
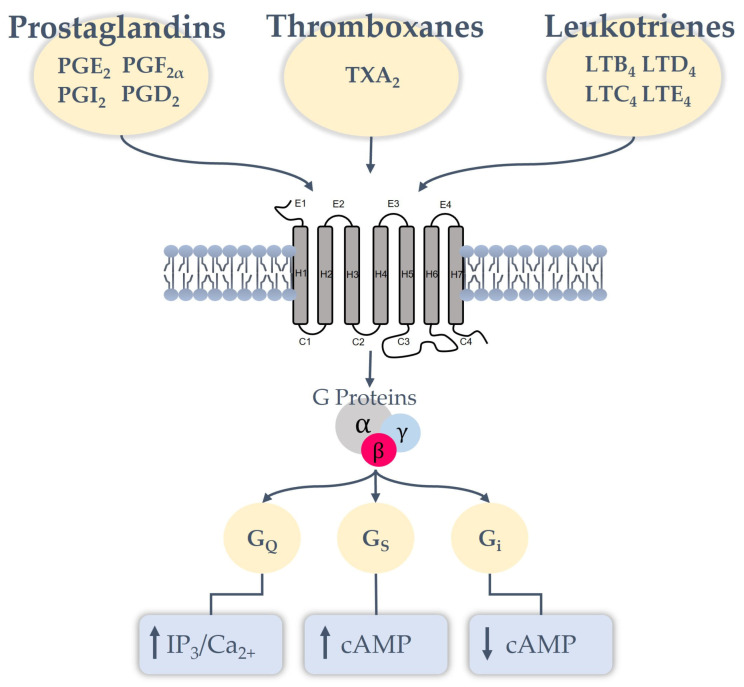
Eicosanoid biosynthesis and GPCR-mediated signalling pathways. Prostanoid receptors mediate the biological effects of PG, LT, and TX and include receptors for PGE_2_, PGF_2_α, PGI_2_, PGD_2_, and TXA_2_, as well as LTB_4_, LTC_4_, LTD_4_, and LTE_4_. These receptors are predominantly integral membrane proteins characterized by a seven-transmembrane (heptahelical) structure and belong to the family of G protein-coupled receptors (GPCRs). Prostanoid receptors display considerable diversity in their G-protein coupling profiles, which leads to distinct downstream physiological responses. Activation of Gq-coupled receptors result in increased intracellular levels of IP_3_ and Ca^2+^, whereas receptors coupled to Gs proteins stimulate cAMP production. In contrast, Gi-coupled receptors inhibit adenylate cyclase activity, leading to reduced intracellular cAMP levels. Prostaglandin E2 (PGE_2_); Prostaglandin F2α (PGF_2_α); Prostaglandin I2 (PGI_2_); Prostaglandin D2 (PGD_2_); Thromboxane A2 (TXA_2_); Leukotriene B4 (LTB_4_); Leukotriene C4 (LTC_4_); Leukotriene D4 (LTD_4_); Leukotriene E4 (LTE_4_); Cyclic adenosine monophosphate (cAMP); Inositol trisphosphate (IP_3_); Calcium ion (Ca_2+_); Stimulatory G-protein (G_S_); Glutamine/Calcium-binding G protein (G_Q_); Inhibitory G-protein (G_i_). ↑: increase; ↓: decrease.

The biological effects of eicosanoids are mediated through their interaction with specific receptors expressed in different cells and tissues. Most eicosanoid receptors belong to the class A (type 1) G protein-coupled receptor (GPCR) family and are characterized by a seven-transmembrane helical structure. These receptors exhibit high specificity and variable affinity for lipid mediators, primarily triggering local autocrine and paracrine responses [10]. Eicosanoid GPCRs are characterized by their coupling to various heterotrimeric G proteins, enabling a single receptor to activate multiple downstream signalling cascades. Based on ligand specificity and selective interaction with stimulatory G protein alpha subunit (Gαs), Inhibitory/Other G protein alpha subunits (Gαi/o), G protein alpha q and alpha 11 subunits (Gαq/11), or G protein alpha 12 and alpha 13 subunits (Gα12/13), receptor activation may lead to different intracellular responses, including modulation of cyclic adenosine monophosphate (cAMP) levels, intracellular Ca^2+^ mobilization, and kinase-dependent signalling pathways [10]. Among the best-characterized classes are prostanoid receptors, which mediate the biological effects of prostaglandins, prostacyclins, and thromboxanes. These receptors include those for PGD_2_ (D-prostanoid receptor 1 (DP1) and D-prostanoid receptor 2 (DP2)), PGE_2_ prostaglandin E2 receptor 1 (EP1) - EP4, PGF_2_α prostaglandin F (FP), PGI_2_ (prostaglandin I₂ (IP)), and TXA_2_ thromboxane prostanoid (TP). Prostanoid receptors display substantial heterogeneity in G-protein coupling and downstream signalling. IP, DP1, Prostaglandin E receptor 2 (EP2), and EP4 receptors preferentially couple to Gαs proteins, promoting intracellular cAMP accumulation, whereas EP1, FP, and TP mainly signal through Gαq pathways, resulting in increased intracellular Ca^2+^ levels. In contrast, specific EP3 isoforms are associated with Gαi proteins and inhibit cAMP production (Figure 1). This receptor heterogeneity enables prostanoids to exert context-dependent and sometimes opposing biological effects within the same tissue [21]. Another major receptor family includes leukotriene receptors, which recognize mediators generated through the LOX pathway. leukotriene B4 receptor 1 (BLT_1_) and leukotriene B4 receptor 2 (BLT_2_) receptors mediate the effects of LTB_4_, whereas cysteinyl leukotrienes-1 (CysLT_1_) and cysteinyl leukotrienes-2 (CysLT_2_) respond to cysteinyl leukotrienes such as LTC_4_, LTD_4_, and LTE_4_. These receptors are critically involved in inflammatory and immune responses, particularly in leukocyte recruitment and cytokine signalling [22]. In addition to prostanoid and leukotriene receptors, the eicosanoid signalling system also includes receptors involved in the resolution phase of inflammation. For example, lipoxin A4 receptor/formyl peptide receptor 2 (ALX/FPR2) binds LXA_4_ and other specialized pro-resolving mediators, promoting anti-inflammatory and pro-resolving responses [23]. Further complexity within the eicosanoid signalling network is provided by non-canonical GPCRs, including oxoeicosanoid receptor 1 (OXE1), as well as receptors such as G protein-coupled receptor 32 (GPR32) and chemerin receptor 23 (ChemR23), which interact with ω-3 derived lipid mediators and resolvins [24]. Overall, the diversity of eicosanoid receptors highlights the complexity of lipid-mediated signalling and explains how structurally related mediators can induce distinct biological effects depending on receptor expression, G-protein coupling, and cellular context. This receptor-level specificity represents an important determinant of both physiological regulation and pathological processes, making eicosanoid GPCRs promising pharmacological targets [24]. Moreover, several studies have demonstrated the expression of eicosanoid-synthesizing enzymes and specific prostanoid and leukotriene receptors within cardiomyocytes and myocardial tissue, supporting the existence of a tightly regulated interaction between arachidonic acid-derived mediators and cardiac physiology. These signalling pathways are involved in the regulation of myocardial remodelling, calcium homeostasis, inflammatory responses, and cardiomyocyte survival, further highlighting the relevance of eicosanoid signalling in cardiomyopathy and heart failure progression [24].

#### Role of Eicosanoid Receptors in Cardiomyopathy and HF Progression: Molecular Mechanisms and Preclinical Evidence

The primary impacts of PG and isoprostanes involve vascularization, platelets, and leukocytes in the setting of cardiovascular balance [25]. Haemostatic responses are mediated by prostanoid receptors, which cause vasoconstriction, mainly via TXA_2_; similar effects are produced by TxB2 and PGF2α, and these are reversed by vasodilation caused by PGE_2_, PGI_2_, LTC_4_, LTD_4_, and LTE_4_ [26]. In addition, exogenous PGE_2_ has been shown to reduce cardiac contractility in both isolated beating hearts and individual adult ventricular myocytes via its EP3 receptor subtype, while the stimulation of the EP4 produced opposing effects [27]. In particular, PGE_2_ exhibits equal binding affinity to EP3 and EP4, and consequently, the outcome of PGE_2_ depends on the expression levels of these receptors [28]. Additionally, PGE_2_ also affects calcium handling in cardiac myocytes by regulating phospholamban (PLN) phosphorylation, thereby modulating relaxation and contractility [29]. Indeed, PLN controls calcium ion release from and reuptake into the sarcoplasmic reticulum (SR), contributing to proper cardiac muscle function [30].

On these physiological bases, it becomes evident that the major cardiovascular risk factors can profoundly alter these mechanisms. Hypertension alters the balance between prostacyclin and vasoconstrictive eicosanoids, favouring vascular contraction and platelet aggregation through receptor-level genetic changes [31,32]. For the first time, a recent study showed that an EP3 antagonist blocked cardiac dysfunction and blood pressure increase. Additionally, the findings indicate that mice with EP3 overexpression in their heart muscle have impaired baseline cardiac function, declining heart function when given angiotensin II (ANG II), and higher levels of inflammatory substances in their left ventricle. These results demonstrate that an EP3 antagonist blocked heart dysfunction development with ANG II and significantly reduced the blood pressure rise [32]. Moreover, mice with extra EP3 expression in cardiomyocytes showed impaired heart function at baseline, worsened cardiac function following ANG II exposure, and elevated inflammatory molecules in the left ventricle [32].

This vulnerability is further compounded in diabetes, where oxidative stress enhances isoprostane formation and EP3 receptor expression, lowering intracellular cAMP and promoting platelet activation. In addition, dyslipidemia contributes to lipid peroxidation, which in turn stimulates the synthesis of pro-aggregating eicosanoids, while smoking exacerbates oxidative stress and activates receptors such as the TP and CysLT [33]. Obesity, through adipocyte-derived PGE_2_, triggers EP3 receptor signalling, fostering inflammation and metabolic dysfunction. Collectively, these cardiovascular risk factors profoundly reshape eicosanoid synthesis and receptor activity, impairing vascular homeostasis and enhancing platelet and inflammatory responses [34]. The resulting dysregulation drives a prothrombotic and vasoconstrictive milieu, ultimately sustaining chronic vascular inflammation [35].

When myocardial infarction (MI) occurs, PGE_2_ production and EP3/EP4 receptor expression increase [36]. Cardiac hypertrophy is an adaptive response post-MI to compensate for increased workload and stabilize contractile function. It is characterized by increased expression of transcription factors, natriuretic peptides, brain natriuretic peptide and atrial natriuretic peptide (Myh7), and growth factors. Regarding MI, PGE_2_ may exert both protective and detrimental effects depending on receptor involvement and cell type, as the same receptors can trigger distinct cellular responses [31]. In particular, PGE_2_, through its interaction with the EP4 receptor, blocks β-adrenergic stimulation of cardiac myocytes, thereby preventing the contractile dysfunction observed in HF. In addition, the PGE_2_/EP4 pathway inhibits transforming growth factor beta 1 (TGF β1) production and secretion in cardiac cells, protecting against cardiomyocyte hypertrophy and cardiac fibrosis by limiting collagen production in fibroblasts [37]. EP4 overexpression was also found to improve heart function after MI by reducing pro-inflammatory cytokines and collagen accumulation, resulting in increased EF and reduced macrophage infiltration [38]. Furthermore, EP4 signalling stimulates cardiomyocyte hypertrophy during MI, whereas selective EP4 deletion in cardiomyocytes attenuates hypertrophy and remodelling, while global cardiac EP4 deletion exacerbates infarct size [38]. Therefore, although the PGE_2_/EP4 axis appears to exert dual effects in myocardial ischemia, its cardioprotective role seems to prevail [38].

Conversely, EP3 signalling appears to negatively affect HF progression, although some evidence suggests a protective role against reperfusion injury and infarct size reduction [31]. These findings suggest that EP3 signalling may exert context-dependent effects depending on the pathological setting and duration of activation. While chronic EP3 activation appears to promote inflammation, hypertrophy, and contractile dysfunction, transient activation during acute ischemic injury may exert protective effects by limiting acute cellular damage. Increased EP3 expression decreases cardiomyocyte contraction, promotes hypertrophy, and activates myocyte enhancer factor 2 (MEF2), contributing to cardiac remodelling and HF development [39]. EP3 overexpression following HF is associated with reduced EF, left ventricular enlargement, impaired cardiomyocyte contractility, increased collagen deposition, and macrophage infiltration [39]. Using a novel mouse model, researchers demonstrated that cardiomyocyte-specific EP3 deletion improved cardiac function in both male and female mice and prevented hypertrophy and collagen accumulation after MI. Moreover, delayed treatment with the EP3 antagonist significantly improved EF and shortening fraction, supporting the potential therapeutic role of EP3 antagonists in HF [36]. Although these compounds have not yet been clinically investigated in HF, another EP3 antagonist, DG 041, has already been tested in vivo in models of diet-induced obesity, type 2 diabetes (T2D), and thrombosis [36].

Increased PGE_2_, an inflammatory lipid, correlates with decreased heart function in both obesity and T2D. In this context, modulation of EP3 and EP4 receptors, the main targets of PGE_2_, has emerged as a promising strategy to improve cardiac contractility and myocardial function. In db/db mice, a widely used T2D model, two weeks of systemic EP3 antagonist treatment (20 mg/kg) improved EF and fractional shortening without affecting heart rate. These findings indicate that EP3 blockade and EP4 activation enhance cardiomyocyte contractility and calcium cycling under both normoglycaemic and hyperglycaemic conditions, as demonstrated by increased Ca^2+^ transient amplitude and shortened time to peak and decay [27]. These data further support the detrimental role of EP3 overexpression in cardiomyocyte dysfunction, whereas EP4 signalling appears to exert cardioprotective effects. However, since gene expression analyses were performed on whole-cardiac-tissue, the observed changes may also derive from fibroblasts or endothelial cells, thus requiring further investigations on isolated cardiomyocytes [27].

Mechanistically, EP3 and EP4 signalling exert opposite effects through modulation of intracellular cAMP levels and PLN phosphorylation. EP3 activation inhibits PLN phosphorylation, reducing calcium reuptake and contractility, whereas EP4 activation enhances PLN phosphorylation, thereby improving calcium dynamics and myocardial contraction [29]. Moreover, MI induces upregulation of both EP3 and EP4 receptors, although EP3 expression appears to predominate, suggesting that inflammatory and ischemic conditions preferentially activate the PGE_2_/EP3 axis, ultimately contributing to cardiac dysfunction [29,40].

Further evidence supporting the beneficial effects of EP4 derives from studies in prostaglandin E receptor 4 knockout (EP4 KO) mice and murine ventricular cardiomyocytes, where EP4 deletion was associated with reduced expression of electron transport chain proteins, decreased ATP production, impaired antioxidant activity, and increased reactive oxygen species (ROS) generation. These findings suggest that loss of EP4 signalling contributes to mitochondrial dysfunction and impaired myocardial contractility, whereas EP3 signalling may promote cardiac dysfunction [41].

In parallel, PGE_2_ has been identified as a potent activator of the MEF2 transcription factor in cardiomyocytes, predominantly through EP3 receptor signalling. Mechanistically, this process involves the Protein Kinase D (PKD)-mediated/Rac1/HDAC5 signalling pathway (Rac1-HDAC5), promoting hypertrophic gene transcription and linking myocardial inflammation to adverse remodelling and HF progression [42]. Conversely, EP4 signalling appears to activate alternative cAMP-dependent pathways distinct from β-adrenergic signalling, further supporting its protective role in the myocardium [42].

Additional studies demonstrated that EP4 overexpression protects against MI by improving cardiac function and reducing inflammation in the left ventricle. Moreover, both PGE_2_ and EP4 agonists reduce monocyte chemotactic protein-5 (MCP-5) secretion and pro-inflammatory signalling in cardiac fibroblasts, suggesting anti-inflammatory paracrine effects within the myocardium [43]. Despite uncertainties regarding the contribution of endogenous versus exogenous PGE_2_ signalling, these findings support the therapeutic potential of EP4 activation in limiting inflammation and adverse cardiac remodelling [43].

Beyond EP3 and EP4, other prostanoid receptors also contribute to cardiomyopathy progression. Enhanced TXA_2_/TP signalling has been associated with hypertension, vasoconstriction, platelet activation, and early cardiac fibrosis, particularly under conditions of impaired PGI_2_ activity. In prostaglandin I_2_ receptor knockout mice, low-dose aspirin attenuated blood pressure elevation and prevented fibrosis by selectively reducing TXA_2_ synthesis [44]. Furthermore, activation of the PGD_2_ receptor CRTH2 promotes cardiomyocyte apoptosis through the Gαq-Ca^2+^-m-calpain-caspase-12 pathway in myocardial ischemia and doxorubicin-induced cardiotoxicity (DIC), whereas genetic or pharmacological CRTH2 inhibition improves cardiac function and reduces myocyte loss [40]. Emerging evidence suggests that sex-related differences may significantly influence eicosanoid signalling and the onset and progression of cardiomyopathy. Sex hormones, particularly estrogens, are known to modulate AA metabolism, COX and LOX activity, prostanoid biosynthesis, and inflammatory signalling pathways [45]. These mechanisms may contribute to sex-specific differences in oxidative stress, endothelial dysfunction, myocardial inflammation, fibrosis, and ventricular remodelling. In particular, estrogens appear to promote vasoprotective and anti-inflammatory effects partly through the regulation of prostacyclin and thromboxane balance, whereas dysregulation of these pathways may increase cardiovascular susceptibility and adverse cardiac remodelling. Furthermore, sex-dependent differences in immune responses and platelet activation could also influence the progression of HF and the response to therapies targeting eicosanoid pathways [44]. Despite these observations, most currently available preclinical studies investigating eicosanoid-mediated cardiac dysfunction were predominantly conducted in male animals, limiting the understanding of female-specific mechanisms and reducing the translational relevance of these findings. Therefore, further studies specifically designed to investigate sex-dependent differences are needed to improve the development of personalized therapeutic strategies in cardiomyopathy and HF [45]. In addition to sex-related differences, aging is increasingly recognized as a major contributor to the development and progression of cardiomyopathy and HF, mainly through mechanisms involving chronic low-grade inflammation, oxidative stress, endothelial dysfunction, and mitochondrial impairment. These age-related alterations may significantly affect AA metabolism and eicosanoid biosynthesis, promoting the accumulation of pro-inflammatory lipid mediators involved in myocardial fibrosis, ventricular remodelling, and progressive cardiac dysfunction [46]. In particular, aging has been associated with an imbalance between protective and detrimental eicosanoids, leading to impaired vascular homeostasis, enhanced inflammatory activation, and reduced myocardial adaptive capacity. Furthermore, inflammation-related alterations in cytochrome P450-mediated AA metabolism may contribute to pathological cardiac hypertrophy and fibrosis through the dysregulation of downstream signalling pathways involved in oxidative stress and extracellular matrix deposition [47]. These mechanisms may also influence the progression of age-related HF and modify the response to pharmacological therapies targeting inflammatory and lipid-mediated pathways. Despite the growing clinical relevance of aging-associated cardiomyopathy, the precise contribution of eicosanoid signalling to age-dependent cardiac dysfunction remains only partially understood and warrants further investigation. Gene-environment interactions are increasingly recognized as important contributors to the heterogeneity of cardiomyopathy onset, progression, and therapeutic response. Genetic polymorphisms involving enzymes associated with AA metabolism, COX and LOX pathways, prostanoid receptors, and inflammatory mediators may significantly influence individual susceptibility to cardiovascular dysfunction and HF development [48]. In parallel, environmental and lifestyle-related factors, including smoking, obesity, diabetes, dietary habits, physical inactivity, hypertension, and chronic exposure to oxidative stress, can profoundly modulate eicosanoid biosynthesis and inflammatory signalling pathways. These factors may promote the accumulation of pro-inflammatory lipid mediators, endothelial dysfunction, myocardial fibrosis, platelet activation, and adverse ventricular remodelling [49]. Furthermore, the interaction between genetic predisposition and environmental triggers may partly explain the marked interindividual variability observed in disease severity, progression, and response to therapies targeting inflammatory and lipid-mediated pathways. Epigenetic modifications induced by environmental stressors may also contribute to the dysregulation of genes involved in eicosanoid signalling and cardiovascular homeostasis. Although growing evidence supports the relevance of gene–environment interactions in cardiovascular diseases, the precise mechanisms linking these factors to eicosanoid-mediated cardiac dysfunction remain insufficiently explored and deserve further investigation in both experimental and clinical settings [50]. Table 1 summarizes the results regarding the role of the eicosanoids and their receptors in cardiomyopathy onset and progression.

**Figure 2:**
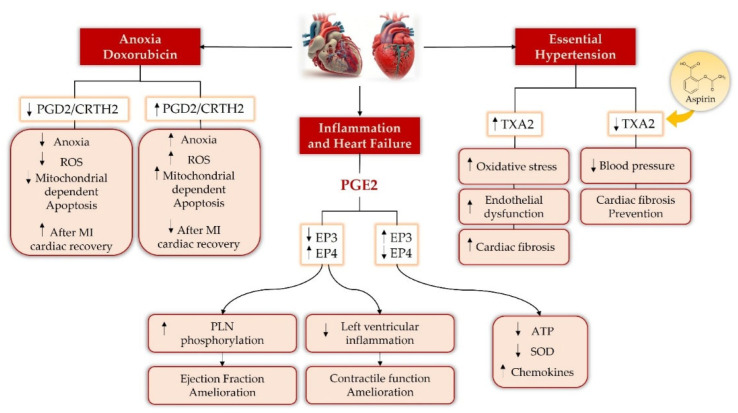
Schematic representation of the main molecular pathways involved in cardiac dysfunction in various pathological conditions. In the presence of anoxia and treatment with doxorubicin, marked activation of the PGD_2_/CRTH2 axis is observed in cardiomyocytes, resulting in oxidative stress accumulation and apoptosis induction. In inflammation and HF models, the increased expression of PGE_2_ and its interaction with EP3 and EP4 receptors determine different responses: both EP3 inhibition and EP4 activation could promote PLN protein phosphorylation, improving intracellular calcium regulation and reducing left ventricular inflammation, thus contributing to improved contractile function. On the contrary, both EP3 up-regulation and EP4 down-regulation could promote ATP-dependent pathway inhibition, determining ROS production and the reduction of scavenger enzymes; this, in turn, could induce chemokines overexpression and activation. In essential hypertension, increased systemic biosynthesis of TxA_2_ is associated with oxidative stress, increased blood pressure, and cardiac fibrosis development. A selective reduction in TXA_2_ synthesis, achieved by administering low-dose aspirin, can limit the increase in blood pressure, thus preventing the early onset of cardiac fibrosis. Abbreviations: PGD_2_: Prostaglandin D_2_; CRTH2: Chemoattractant Receptor-Homologous molecule expressed on Th2 cells; MI: myocardial infarction; PGE_2_: Prostaglandin E_2_; EP3: Prostaglandin E receptor 3; EP4: Prostaglandin E receptor 4; TxA_2_: Thromboxane A_2_; ATP: Adenosine Triphosphate; PLN: Phospholamban; SOD: Superoxide Dismutase. ROS: Reactive Oxygen Species. ↑: increase; ↓: decrease.

Lipid mediators’ role in activating signalling pathways, through receptor interactions and distribution, significantly contributes to cardiomyopathy Figure 2.

## 2. Beneficial Effects of Natural Derivatives in Cardiomyopathy

Based on current guidelines, a personalized, multidimensional therapeutic strategy for cardiomyopathy is recommended. This strategy is influenced by the classification of the cardiomyopathy, symptom severity, and the presence of genetic or acquired risk factors [51]. Cardiomyopathy treatment typically combines pharmacological strategies, aimed at controlling symptoms and improving cardiac function, with lifestyle interventions, non-drug approaches, and cardiac rehabilitation programmes [51]. Although these strategies provide significant prognostic benefits, their long-term use may be associated with adverse effects, prompting interest in complementary approaches with broader mechanisms of action and more favourable safety profiles [52]. Particularly, treatment with anthracyclines can result in heart failure in 15–17% of patients, associated with mitochondrial dysfunction, ROS accumulation, calcium dysregulation, cardiomyocyte structural deterioration, and apoptotic cell death [53]; these adverse effects highlight the need for adjunctive therapies capable of targeting multiple molecular mechanisms underlying cardiac injury. In this context, natural products and nutraceuticals, including omega-3 fatty acids, polyphenols, phytosterols, Coenzyme Q10 (CoQ10), and vitamins, have attracted attention for their capacity to modulate key cardiovascular pathophysiological processes, exerting antioxidant, anti-ischaemic, anti-proliferative, hypotensive, antithrombotic, and cholesterol-lowering activities [52].

Icariin (ICA), a flavonoid from the *Epimedii herba* species, demonstrates potent cardioprotective effects in experimental models of DOX-induced cardiac injury. In preclinical studies, ICA reduces oxidative stress, prevents mitochondrial dysfunction, and counteracts apoptosis in cardiomyocytes. By inhibiting phosphodiesterase 5A (PDE5a), it further demonstrates its capacity to influence ROS-mediated signalling and protect cardiac function. The observed combined effects imply that ICA offers cardiac protection by concurrently addressing several mechanisms involved in drug-induced cardiotoxicity, such as cardiomyocyte structural decline and programmed cell death [53,54].

Ferutinine, a bioactive compound derived from *Ferula communis* L., exhibits antioxidant, anti-inflammatory, and anti-proliferative properties in vitro. In rat embryonic cardiomyoblast cell line (H9C2), ferutinine significantly reduces H_2_O_2_-induced ROS production, mitigating oxidative stress and preventing early signs of cellular hypertrophy. Dose-dependent studies indicate that even low concentrations effectively attenuate ROS accumulation, highlighting its potential as a cardioprotective agent that modulates oxidative stress pathways and limits maladaptive cardiac remodelling [55].

Bergamot (*Citrus bergamia*) contains a rich polyphenolic profile in its juice and albedo, including flavonoids and glycosides with antioxidant and anti-inflammatory properties. Experimental models of DIC have shown that the bergamot polyphenolic fraction (BPF) restores autophagy, reduces cardiomyocyte apoptosis, and mitigates reactive hypertrophy. These effects are associated with modulation of cellular stress pathways and suppression of pro-fibrotic signalling, suggesting that BPF can protect cardiac structure and function under oxidative conditions [56].

*Cynara cardunculus* L. extracts have similarly demonstrated cardioprotective activity in experimental settings, primarily through antioxidant and anti-inflammatory mechanisms. By reducing ROS accumulation and dampening inflammatory signalling, these extracts prevent cardiomyocyte structural damage and reduce fibrosis development. The key role of *Cynara* compounds in the modulation of intracellular stress and survival pathways further supports their potential as natural multi-target cardioprotective agents [57].

Resveratrol, a stilbene polyphenol found in grapes and red wine, has demonstrated significant cardioprotective effects in experimental models of diabetic cardiomyopathy. In streptozotocin-induced diabetic rats, resveratrol improves mitochondrial function by enhancing the deacetylation of Peroxisome proliferator-activated receptor gamma coactivator 1-alpha (PGC-1α) via activation of Sirtuin 1 (SIRT1), leading to improved mitochondrial biogenesis and energetic efficiency. This modulation reduces oxidative stress, preserves cardiomyocyte viability, and attenuates cardiac dysfunction characteristic of diabetic cardiomyopathy. Additionally, resveratrol’s effects on key stress and survival signalling pathways help prevent maladaptive remodelling and functional decline, highlighting its potential as a multi-target cardioprotective nutraceutical [58].

Quercetin, a dietary flavanol abundant in onions, apples, berries, and tea, has revealed notable cardioprotective activity in experimental models of cardiomyopathy, particularly diabetic cardiomyopathy. In streptozotocin-induced diabetic mouse models, quercetin administration significantly improved cardiac contractile function (e.g., ejection fraction and fractional shortening) and attenuated pathological remodelling by reducing myocardial inflammation, fibrosis, and dysregulated glycerophospholipid metabolism. Mechanistically, these benefits are linked to quercetin’s potent antioxidant and anti-inflammatory properties, suppression of pro-inflammatory mediators, and modulation of metabolic pathways that underlie cardiomyocyte stress responses. The wide range of effects suggests quercetin’s potential to counteract major pathological mechanisms in cardiomyopathy, highlighting its promise as a natural agent for cardioprotection in oxidative and metabolic cardiac injury [59].

Curcumin, a polyphenolic constituent of *Curcuma longa* L., widely used as a dietary spice, displays significant cardioprotective activity in models of diabetic cardiomyopathy. In streptozotocin-induced diabetic rats and high-glucose-treated cardiomyocytes, curcumin enhances AKT-mediated nuclear translocation of nuclear factor erythroid 2-related factor 2 (Nrf2), upregulating antioxidant effectors such as heme oxygenase-1 (HO-1) and glutamate cysteine ligase (GCL), which collectively boost cellular defence against oxidative stress and mitochondrial damage. By activating the Nrf2/antioxidant response element (ARE) axis and curbing ROS accumulation, curcumin also attenuates pyroptosis, a pro-inflammatory form of programmed cell death implicated in diabetic cardiac injury. These molecular actions translate into reduced cardiomyocyte death and myocardial fibrosis, thereby preserving cardiac structure and function in diabetic cardiomyopathy models [60].

Astaxanthin (ASTA), a xanthophyll carotenoid obtained from *Haematococcus pluvialis*, shows potent antioxidant, anti-inflammatory, and anti-apoptotic activity. In preclinical models of cardiomyopathy, ASTA supplementation improves cardiac function, restores redox balance, and positively influences lipid and glucose metabolism. These effects collectively reduce cardiomyocyte stress and prevent structural deterioration, highlighting ASTA as a promising natural compound capable of modulating multiple pathways involved in cardiac injury [61].

*Cyclocarya paliurus*, a deciduous tree native to southern China, demonstrates cardioprotective effects in diabetes mouse models. Ethanolic extracts reduce cardiac fibrosis, attenuate structural damage, suppress inflammatory responses, and inhibit apoptosis. Cell survival is promoted by the activation of the Phosphoinositide 3-kinase/ak strain transforming (PI3K/Akt) signalling pathway, and inflammation is regulated by NF-κB, which is inhibited, leading to these effects. The multiple therapeutic targets indicate that *Cyclocarya paliurus* may be effective in treating diabetic cardiomyopathy [62].

*Capparis spinosa* L. exhibits promising cardioprotective effects in diabetic cardiomyopathy, mainly due to its polyphenol-rich extract (CSN-50%). This fraction shows strong antioxidant activity, comparable to ascorbic acid, and significantly reduces ROS production and cardiomyocyte apoptosis in vitro. In vivo, it lowers blood glucose levels, attenuates cardiac fibrosis, and improves myocardial function. These effects are likely mediated by multi-target mechanisms involving oxidative stress and apoptosis pathways, although specific molecular targets such as Nrf2 and PI3K/Akt require further validation [63].

*Phoenix dactylifera* L. (date palm), rich in phenols, flavonoids, and carotenoids, exerts cardioprotective effects in diabetic cardiomyopathy. Experimental evidence shows that its extracts might enhance insulin signalling and improve glucose lipid metabolism, while directly inhibiting TGF-β-mediated pro-fibrotic pathways and fibroblast activation. The dual action of reducing fibrosis and hypertrophy maintains cardiac structure and function, highlighting the multifaceted mechanisms by which date palm phytocompounds provide protection [64].

Green tea (*Camellia sinensis* L.) is a widely consumed source of catechins with strong antioxidant and anti-inflammatory properties. In models of chronic kidney disease, supplementation with green tea extract attenuates left ventricular hypertrophy by reducing cardiomyocyte enlargement and oxidative stress. These effects highlight the ability of green tea polyphenols to modulate molecular pathways underlying maladaptive cardiac remodelling and ventricular dysfunction onset and development [65].

*Zingiber officinale* (ginger), a medicinal and culinary plant, contains bioactive compounds such as gingerols and shogaols with antioxidant, anti-inflammatory, and cardioprotective properties. In experimental models of pressure-overload-induced cardiac stress, ginger extract significantly attenuates left ventricular hypertrophy, reduces myocardial fibrosis, and preserves systolic function. The observed effects correlate with altered cellular stress signalling, including decreased pro-hypertrophic pathways and lower accumulation of ROS, thereby mitigating detrimental cardiac remodelling. These findings highlight the potential of ginger as a natural multi-target agent for preventing or mitigating pathological cardiac growth and dysfunction [66].

*Andrographis paniculata*, a medicinal herb traditionally used in Asian ethnomedicine, contains diterpenoids such as andrographolide with potent antioxidant and anti-inflammatory properties. Recent in silico and in vitro research has shown that ethanolic extracts of *A. paniculata* can attenuate cardiac hypertrophy by modulating key molecular pathways involved in inflammation and oxidative stress. Specifically, extract treatment reduced hypertrophic markers in angiotensin II-treated H9C2 cardiomyoblasts, associated with upregulation of the Nrf2 antioxidant pathway and downregulation of NF-κB and NLR family pyrin domain containing 3 (NLRP3) inflammasome signalling, thereby suppressing pro-inflammatory gene expression and ROS production. These findings support a multi-target cardioprotective role for *Andrographis paniculata* in the context of pathological cardiac growth and remodelling [67].

*Allium sativum* L. (garlic), a well-known culinary and medicinal plant, contains organosulfur compounds such as allicin and alliin, which are recognized for their significant antioxidant and cardioprotective properties. In vitro studies conducted on isolated adult rat cardiomyocytes demonstrate that garlic skin and flesh extracts significantly inhibit norepinephrine-induced hypertrophy and apoptosis, while also mitigating oxidative stress. These protective effects are partially mediated by enhanced production of nitric oxide (NO) and hydrogen sulfide (H_2_S), which can modulate redox balance and signalling pathways linked to cellular growth and survival. By reducing ROS levels and limiting pro-hypertrophic and apoptotic signalling, garlic extracts show multi-target modulation of key mechanisms implicated in pathological cardiac remodelling, underscoring their potential role in mitigating cardiomyocyte enlargement and injury [68].

Lotus bee pollen extract, derived from the pollen collected by bees on lotus plants, is rich in flavonoids, phenolic acids, and other bioactive constituents that confer antioxidant, anti-inflammatory, and anti-apoptotic properties [69]. In an in vitro model of isoproterenol-induced cardiomyocyte hypertrophy using H9c2 cells, lotus bee pollen extract dose-dependently reduced cell enlargement, decreased malondialdehyde (MDA) levels, and increased activities of scavenger enzymes such as superoxide dismutase (SOD) and glutathione (GSH). At the molecular level, the extract mitigated hypertrophic markers and pro-inflammatory cytokine expression while enhancing the B-cell lymphoma/leukemia-2/Bcl-2-associated X protein (Bcl-2/Bax) ratio, indicating inhibition of apoptotic signalling. Bioinformatics and molecular docking analyses, supported by Western blot validation, suggest that Lotus bee pollen extract’s protective effects involve suppression of the Janus Kinase 2/Signal Transducer and Activator of Transcription 3 (JAK2/STAT3) signalling pathway, a key mediator of pathological cardiomyocyte growth. These findings underscore the multi-target cardioprotective potential of lotus bee pollen extract through modulation of hypertrophic signalling [70]. These effects may also indirectly modulate eicosanoid-mediated inflammatory signalling associated with cardiac remodelling.

*Olea europaea* L. extract (olive leaf extract), derived from the leaves of the olive tree, is rich in phenolic compounds such as oleuropein and has been shown to exert cardioprotective effects in a streptozotocin-induced model of diabetic cardiomyopathy [65]. In diabetic rats, supplementation with olive leaf extract significantly attenuated cardiac hypertrophy and interstitial fibrosis, while improving the heart-to-body weight ratio and reducing markers of oxidative stress within the myocardium. These beneficial effects are associated with modulation of redox balance and suppression of pro-fibrotic signalling pathways, which drive maladaptive remodelling, highlighting the ability of olive leaf phenols to counteract structural and functional deterioration in cardiomyopathy. The broad range of actions seen in *Olea europaea* L. extracts highlights their potential to treat cardiomyopathy by reducing fibrosis and pathological growth through antioxidant and anti-remodelling effects [71]. Such mechanisms could contribute to the attenuation of pro-inflammatory eicosanoid production in diabetic cardiomyopathy.

*Achyranthis bidentatae* radix and *Saururus chinensis*, used in Asian traditional medicine, have been shown to exert cardioprotective effects in experimental models of cardiac hypertrophy. Treatment with extracts from these plants significantly attenuated cardiomyocyte enlargement, reducing myocardial fibrosis and suppressing oxidative stress. These findings suggest a connection to altered growth factor signaling, insulin-dependent processes, and inflammatory reactions. Specifically, there’s a decrease in Signal Transducer and Activator of Transcription 3 (STAT3) and Matrix Metalloproteinase-9 (MMP9) activity, both crucial for pathological hypertrophy and extracellular matrix remodelling. These anti-inflammatory properties may further interact with eicosanoid-related pathways involved in myocardial fibrosis and remodelling. This suggests the potential of *Achyranthis bidentatae* and *Saururus chinensis* as natural multi-target interventions to limit hypertrophic growth and preserve cardiac structure and function [72].

**Figure 3:**
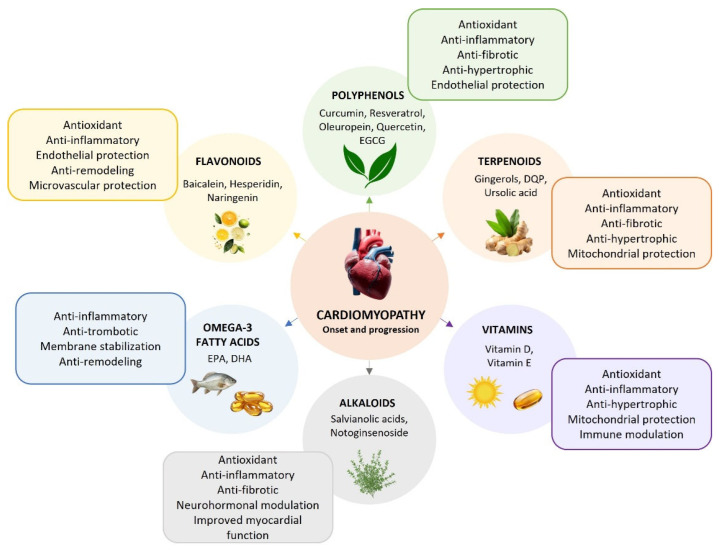
Natural derivatives with cardioprotective potential in cardiomyopathy. Polyphenols, flavonoids, terpenoids, omega-3 fatty acids, vitamins, and alkaloids exert multiple biological effects through antioxidant, anti-inflammatory, anti-fibrotic, anti-hypertrophic, anti-thrombotic, and mitochondrial protective mechanisms. These compounds contribute to the modulation of myocardial remodelling, inflammatory signalling, cardiomyocyte survival, and cardiac function preservation. Eicosapentaenoic acid (EPA); docosahexaenoic acid (DHA); DanQi Pill (DQP); epigallocatechin gallate (EGCG).

The analysed studies highlight the pleiotropic cardioprotective potential of natural compounds, which act through complementary mechanisms to counteract oxidative stress, inflammation, apoptosis, and metabolic disturbances (Table 2). These multifaceted actions are significant as potential adjuncts to conventional therapies, particularly for conditions such as chemotherapy-induced cardiotoxicity and diabetic cardiomyopathy. These pathways are central to inflammation and cardiac function regulation, thereby presenting a novel therapeutic way to prevent and counteract cardiomyopathy (Figure 3). Among the mechanisms underlying these cardioprotective effects, modulation of eicosanoid biosynthesis and signalling pathways has emerged as a particularly relevant therapeutic target.

### Natural Derivatives and Eicosanoid Signalling Modulation in Cardiomyopathy

Beyond their general antioxidant and anti-inflammatory properties, several natural compounds exert cardioprotective effects through the modulation of eicosanoid-mediated signalling pathways involved in cardiomyopathy progression [73]. In particular, natural derivatives may influence eicosanoid biosynthesis, AA metabolism, and prostanoid receptor signalling by regulating COX and LOX pathways [73]. These effects contribute to the reduction of pro-inflammatory prostaglandins and leukotrienes involved in cardiac inflammation, oxidative stress, fibrosis, hypertrophy, and adverse cardiac remodelling. Moreover, some phytochemicals may indirectly affect calcium homeostasis, mitochondrial function, and inflammatory cytokine production through the modulation of lipid mediator signalling, further supporting their potential therapeutic role in cardiomyopathy and HF [74]. At the molecular level, natural compounds may interfere with eicosanoid signalling through multiple mechanisms, including inhibition of PLA2-mediated AA release, suppression of COX-2 and 5-LOX expression, and modulation of downstream G protein-coupled prostanoid receptors. These effects may attenuate the activation of pro-inflammatory signalling cascades such as nuclear factor kappa B (NF-κB), mitogen-activated protein kinase (MAPK), and TGF-β-related pathways, which are critically involved in cardiac fibrosis, hypertrophy, and ventricular remodelling [74]. Modulating eicosanoid signalling is a promising strategy for harnessing the cardioprotective benefits of natural compounds. Derived from polyunsaturated fatty acids, eicosanoids are key players in inflammation, oxidative stress, and vascular homeostasis, and their improper regulation is significantly connected to the advancement of cardiomyopathies. Investigating the impact of specific phytochemicals on these pathways might reveal innovative therapeutic approaches for cardiac dysfunction [75]. The way their various actions interact with the molecular basis of HF advancement suggests they might serve as an adjunct to conventional therapies. Owing to their multitarget activity on inflammatory and lipid mediator pathways, several Traditional Chinese Medicine (TCM)-derived formulations have also been investigated as complementary strategies for chronic HF management [76]. Ventricular remodelling, myocardial fibrosis, and endothelial dysfunction are all consequences of chronic inflammation, a key factor in cardiomyopathy onset and development. In this scenario, modulation of the AA cascade and its eicosanoid derivatives, PG, TX, and LT, is crucial. DanQi Pill (DQP), a formulation from Salvia Miltiorrhiza and Panax Notoginseng (PNS), represents an example of multitarget phytotherapy. The primary active compounds in DQP are salvianolic acids and saponins from PNS; these work by activating liver X receptor α (LXRα) and suppressing the pro-inflammatory transcription factor NF-κB, thereby exhibiting anti-atherosclerotic effects [77]. DQP has been found to significantly alter AA metabolism at the molecular level; this leads to the downregulation of COX-1, COX-2, LTB4, and leukotriene B4 receptor (LTB4R), as well as PLA2. The profile indicates an effect comparable to dual COX/LOX inhibitors, which might decrease the gastrointestinal toxicity linked to selective COX inhibitors. Moreover, despite not directly impacting TXB2, DQP leads to prostacyclin increase, thus contributing to restoring the dynamic balance between PGI_2_ and TXA2 (P/T ratio), a critical biomarker of thrombotic regulation in HF [76,77]. Furthermore, oxidative stress is a key contributor to myocardial remodelling and the advancement of HF, partly by influencing eicosanoid metabolism. The formation of isoprostanes, particularly 8-iso-prostaglandin F2α (8-iso-PGF2α) from the non-enzymatic oxidation of AA, is not only a solid marker for lipid peroxidation but also a potentially active biological player that can impact vascular tone, inflammation, and endothelial dysfunction [78]. In diabetic cardiomyopathy models, experimental data show vitamin E significantly decreases cardiac 8-iso-prostaglandin F2α, pointing to the potential of antioxidant nutraceuticals to weaken pro-oxidative and pro-inflammatory lipid pathways in heart tissue. The data suggest that nutraceuticals, by influencing redox state, might indirectly impact the eicosanoid network, thus limiting oxidative AA derivatives and preserving myocardial function. Considering this, the interaction of oxidative stress, eicosanoid metabolism, and focused nutritional interventions needs additional investigation as a potential supplementary method for cardiomyopathy management [78].

Clinical evidence further supports the ability of antioxidant nutraceuticals to modulate eicosanoid metabolism. Administering vitamin D and calcium to HF patients with hypovitaminosis D for 14 weeks significantly lowered their plasma 8-isoprostane levels, indicating reduced oxidative stress and inflammation; this also led to better ventricular function. According to this study, targeted nutritional and redox status interventions may indirectly lead to oxidative AA mediator reduction, thereby reinforcing the potential role of nutraceuticals in eicosanoid modulation and myocardial protection [79]. Additionally, a wide range of phenolic and terpenic phytochemicals, characteristic of traditional herbs and spices, have proven to directly impact the enzymes that synthesize eicosanoids. In vitro and preclinical research have shown that curcumin (from Curcuma longa), gingerols (from Zingiber officinale), and capsaicin (from Capsicum) function as dual inhibitors of both COX and 5-LOX. The findings suggest a broad anti-inflammatory action, comparable in mechanism to non-steroidal anti-inflammatory drugs (NSAIDs), with an enhanced safety profile [80]. In addition, several types of flavonoids and polyphenols are known to reduce prostaglandin and leukotriene production by specifically inhibiting COX-2 and downregulating NF-κB, thus combining enzyme activity control with the transcriptional regulation of inflammatory processes [81]. Similarly, acetyl-keto-β-boswellic acid (AKBA), which is derived from Boswellia serrata, has been shown to be a potent inhibitor of 5-LOX and significantly reduce the biosynthesis of pro-inflammatory LT. Baicalein, caffeic acid, and components of Hypericum perforatum are phenolic compounds that have demonstrated the ability to alter the AA cascade. By impacting various points in the eicosanoid metabolic pathway, they contribute to decreased systemic inflammation [82]. Additional findings point to oleuropein (from *Olea europaea* L.) and chlorogenic acid (found in numerous plant foods) as substances that modulate COX and LOX activity, thereby reducing pro-inflammatory mediator production in cellular and experimental models [83].

**Figure 4:**
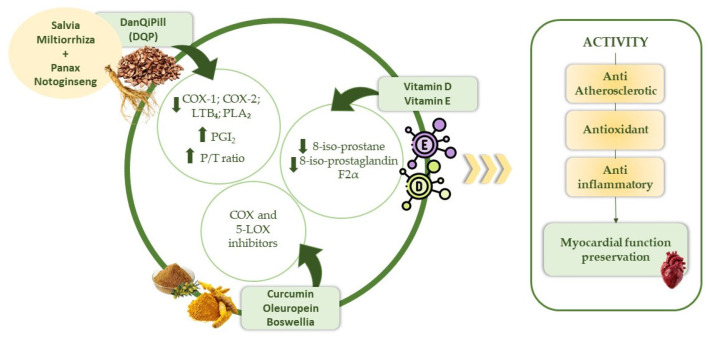
Natural compounds targeting eicosanoid-associated signalling pathways in cardiomyopathy. DanQi Pill (DQP), a formulation derived from *Salvia Miltiorrhiza* and *Panax Notoginseng*, has been reported to downregulate the expression of COX-1, COX-2, LTB_4_, and its receptor (LTB4R), as well as phospholipase A_2_ (PLA_2_). This modulation contributes to restoring the physiological balance between prostacyclin (PGI_2_) and thromboxane A_2_ (TXA_2_), reflected in the normalization of the P/T ratio. In addition, supplementation with vitamins D and E has been shown to significantly decrease myocardial levels of oxidative stress markers such as 8-iso-prostane and 8-iso-prostaglandin F_2_α. Furthermore, natural compounds including curcumin, oleuropein, and *Boswellia*-derived constituents act as dual inhibitors of both cyclooxygenase (COX) and 5-lipoxygenase (5-LOX) in in vitro and preclinical models. The modulation of these pathways exerts beneficial effects, including anti-atherosclerotic, antioxidant, and anti-inflammatory activities, thereby promoting the preservation of myocardial function. DanQi Pill (DQP); Cyclooxygenases-1 (COX-1); Cyclooxygenases-2(COX-2); Leukotriene B4 (LTB_4_); Phospholipase A2 (PLA_2_); Prostaglandin I2 (PGI_2_); prostaglandin I2/thromboxane A2 (P/T); 5-lipoxygenase (5-LOX). ↑: increase; ↓: decrease.

Specific nutraceutical fatty acids, such as dihomo-γ-linolenic acid (DGLA), compete with AA for entry into the same oxygenase enzymes; it has been proven that this promotes the generation of less pro-inflammatory eicosanoids and contributes to a change in the lipid profile, favoring mediators with a lower potential for pro-thrombotic and pro-fibrotic effects. In a similar manner, but with more well-established experimental and clinical support, long-chain omega-3 fatty acids, particularly eicosapentaenoic acid (EPA) and DHA found in fish oil, play a protective role in the cardiovascular system through multiple mechanisms of action [84]. The EPA competes directly with AA for access to COX and other enzymes involved in the biosynthesis of lipid mediators. By converting EPA, these enzymes produce compounds that are less inflammatory and less prone to aggregation compared to AA-derived eicosanoids. The enzymes can also produce molecules that prevent the binding of the aforementioned eicosanoids to their specific receptors. Furthermore, EPA-derived prostacyclin, specifically Prostaglandin I_3_ (PGI3), is as effective as AA-derived PGI2 in stabilizing platelets, whereas EPA-derived thromboxane has no biological activity [85,86]. These results present a unified view where natural compounds influence the eicosanoid pathway through multiple targets, affecting both enzyme-driven synthesis (COX/LOX/PLA2) and the gene expression that controls inflammation (Figure 4). This modulation could potentially reduce chronic inflammation, myocardial fibrosis, thrombotic activation, and ventricular remodelling. Further randomized clinical trials are still needed, however, to define ideal dosages, bioavailability, and cardiovascular endpoint effects. Current findings suggest that using nutraceuticals alongside treatments targeting COX/LOX and inflammatory lipid pathways is a beneficial supplementary approach for eicosanoid regulation and reducing cardiovascular inflammation [87].

## 3. Discussion and Conclusions

This review investigates the role of eicosanoids and their receptors in cardiomyopathy progression, explaining how eicosanoid signalling controls cardiomyocyte metabolism, structural shifts, inflammation, and mitochondrial function amid cardiac metabolic dysfunction. Cardiomyopathy is a complicated condition that, while uncommon, often leads to HF and SCD. The condition’s origin is due to multiple factors, originating from a change in the heart muscle, influenced by genetics, environmental factors, and overall health. Dilated, hypertrophic, restrictive, and arrhythmogenic forms have been progressively characterized [88]. Metabolic and inflammatory diseases are causes, both primary and secondary, of these forms; notably, metabolic cardiomyopathies are connected to obesity, diabetes, and insulin resistance [89]. They are both silent and long-term causes of a drop in heart function. Under these conditions, the heart is unable to efficiently use its energy, causing impaired fat accumulation and cardiac contraction. Eicosanoids are key mediators that come from AA and control many cellular processes, like inflammation, metabolism, and survival [21]. Finally, a recent study examined how EP3, EP4, CRTH2, and TP, as eicosanoid receptors, can be modulated to affect cardiomyocytes, showing that improved contraction function comes from blocking EP3 and activating EP4, which more effectively manages intracellular calcium [90]. These effects have been seen in normal and diabetic models, implying EP3 as a negative target and EP4 as cardioprotective in therapies. Additional studies have shown that EP3 and EP4 directly influence PLN phosphorylation, therefore validating the molecular process connecting lipid signalling to cell mechanics [27]. The CRTH2 receptor’s function has been researched in other studies. This receptor seems to be involved in inducing cardiomyocyte apoptosis during endoplasmic reticulum (ER) stress. Mouse models show that its inhibition enhances recovery after infarction, suggesting CRTH2 is crucial in pro-apoptotic pathways [40]. In contrast, the EP4 receptor has shown benefits in cardiac fibroblasts, decreasing inflammatory chemokines like MCP-5, indicating a paracrine anti-inflammatory effect. Investigations also cover the actions of prostanoids like TXA_2_, impacting vasoconstriction and fibrosis. Research shows that low-dose aspirin therapy can reduce TXA_2_ production and prevent fibrosis, without affecting the levels of related receptors [44]; this proves that a specific therapy can change pro-fibrotic mediators in hypertension or oxidative stress. Regarding intracellular signalling, PGE_2_ activates MEF2, crucial for cardiac remodelling, via pathways converging on HDAC5 inactivation [42]. This link between GPCR_S_ signals and gene transcription emphasizes how eicosanoids precisely control cell fate and cardiac transcription [42]. Furthermore, when the EP4 receptor was deleted in animal models, it led to dysfunctional mitochondria, higher ROS levels, decreased ATP synthesis, and impaired respiratory chain function [43]. This observation provides additional support for the role of the EP4 receptor in modulating contractility and cellular bioenergetics. In a finely regulated system, the activity and distribution of eicosanoid receptors seem to be able to affect how metabolic cardiomyopathy progresses [43]. A comprehensive view of the data suggests that modulating eicosanoid receptors could promote new therapies for preventing and treating metabolic cardiomyopathy. EP3 becomes a key target in disease, while EP4 is a confirmed protector. This study’s findings support future lipid-signalling therapies to shield the heart in complex metabolic dysfunction. Simultaneously, CRTH2 and TP are crucial and demand monitoring in inflammatory behavior, hypertensive stress, and chemotherapy-induced cardiotoxicity. In particular, through the PKC/Nrf2 signalling pathway, the PGE_2_/EP1 axis prevented cardiomyocyte ferroptosis, thus protecting the heart from DIC, and blocking EP1 might be a successful method for preventing and treating chemotherapy-related cardiomyopathy [91]. In parallel with receptor-targeted strategies, evidence increasingly supports the role of natural compounds and nutraceuticals in cardiomyopathy management. A personalized, multidimensional therapeutic strategy for cardiomyopathy is advised per current guidelines. Typical cardiomyopathy treatment involves drug-based approaches for symptom control and cardiac function improvement, complemented by lifestyle adjustments, non-pharmacological methods, and cardiac rehabilitation. Despite the significant prognostic advantages of these strategies, their long-term application might be linked to adverse effects, prompting interest in alternative methods with broader action mechanisms and more favorable safety profiles. Polyphenols, flavonoids, carotenoids, and omega-3 fatty acids have been shown to exert antioxidant, anti-inflammatory, and anti-apoptotic effects, which complement the modulation of eicosanoid pathways [75,81]. These compounds can attenuate oxidative stress, reduce pro-inflammatory eicosanoid derivatives, and restore redox balance, thereby protecting cardiomyocytes and limiting fibrosis, mitochondrial dysfunction, and adverse remodelling. For instance, flavonoids such as quercetin, resveratrol, and curcumin can modulate COX/LOX activity and NF-κB signalling, while long-chain omega-3 fatty acids, including EPA and DHA, compete with AA to produce less pro-inflammatory eicosanoids, ultimately improving cardiac function [84,86]. Collectively, these findings suggest that nutraceuticals may serve as a complementary approach to conventional therapies, targeting both oxidative and lipid-mediated inflammatory pathways to preserve myocardial function in cardiomyopathy. Overall, a better understanding of eicosanoid-mediated mechanisms may open new ways for cardiomyopathy prevention and treatment, ultimately improving patient outcomes and reducing the burden of cardiovascular disease.

## Figures and Tables

**Table 1:** Cardiomyopathy onset and progression: evidence of the crucial role of eicosanoids and their receptor modulation.

Authors, Year	Aim of Studies	Types of Studies Included	Summary of Results	Ref.
Bosma et al., 2022	Examining the effects of blocking EP3 or stimulating EP4 on heart cell function and overall heart performance.	In vitro and in vivo studies	EF and fractional shortening were improved by blocking EP3 and stimulating EP4, regardless of heart rate, under normoglycemic and hyperglycemic states.	[27]
Gu et al., 2016	Evaluation of the acute effects of PGE_2_, sulprostone, and the EP4 agonist on the expression of phosphorylated PLN and sarcoplasmic and endoplasmic reticulum calcium-ATPase 2a (SERCA2a) in adult mouse cardiomyocytes.	In vivo, ex vivo and in vitro studies	Activation of EP3 reduces the phosphorylation of PLN, while activation of EP4 increases it, thereby improving intracellular calcium dynamics and enhancing contractile function.	[29]
Zuo et al., 2018	Examining the apoptotic activity of cardiomyocytes induced by ER stress through the CRTH2 receptor via activation of the m-calpain/caspase-12 pathway.	In vivo and in vitro studies	Activation of the CRTH2 receptor promotes cardiomyocyte apoptosis, while its inhibition improves post-infarction cardiac recovery.	[40]
D’Agostino et al., 2021	Evaluating the impact of administering low-dose aspirin on TXA_2_ levels and hypertension.	In vivo, in vitro and clinical studies	Selective reduction of systemic TXA_2_ biosynthesis with attenuation of blood pressure increase and prevention of early cardiac fibrosis.	[44]
Tóth et al., 2018	Identifying GPCR_S_ mediators that are not yet associated with the activation of the histone deacetylase myocyte enhancer factor 2 (HDAC-MEF2) axis in cardiac myocytes, in order to study the downstream molecular mechanism.	In vivo and in vitro studies	PGE_2_ activates the transcription of the MEF2 factor via two parallel signalling pathways, (Tiam1)/(Rac1)/(PAK2) and (PKD), which converge on the inactivation of the HDAC5.	[42]
Bryson et al., 2024	Identifying the pathways responsible for the development of DCM in male mice with a specific deletion of the EP4 receptor within their cardiomyocytes.	In vivo and in vitro studies	The deletion of the EP4 receptor has been linked to reduced expression of genes which ETC, lower levels of ATP in cardiomyocytes and reduced antioxidant activity of SOD.	[41]
Bryson et al., 2019	Highlighting that PGE_2_ induction, through EP4 receptor activation, reduces inflammation in cardiac fibroblasts by affecting the Ak strain transforming (Akt) and (NF-kB) signalling pathways.	In vitro studies	PGE_2_ inhibits the secretion of MCP-5 in adult mouse cardiac fibroblasts in response to LPS via its EP4 receptor.	[43]

Ak strain transforming (Akt); Adenosine triphosphate (ATP); Chemoattractant receptor expressed on type 2 helper T cells (CRTH2); Diabetic cardiomyopathy (DCM); Ejection fraction (EF); Prostaglandin E2 receptor 3 (EP3); Prostaglandin E2 receptor 4 (EP4); Electron transport chain (ETC); G protein-coupled receptors (GPCR_S_); Histone deacetylase 5 (HDAC5); Histone deacetylase- myocyte enhancer factor 2 (HDAC-MEF2); Lipopolysaccharide (LPS); Monocyte chemotactic protein-5 (MCP-5); Myocyte enhancer factor 2 (MEF2); Nuclear factor kappa-light-chain-enhancer of activated B cells (NF-kB); p21-activated kinase 2 (PAK2); Prostaglandin E_2_ (PGE_2_); Protein kinase D (PKD); Phospholamban (PLN); Ras-related C3 botulinum toxin substrate 1 (Rac1); Sarcoplasmic and endoplasmic reticulum calcium-ATPase 2a (SERCA2a); Superoxide dismutase (SOD); T-cell lymphoma invasion and metastasis 1 (Tiam1); Thromboxane A2 (TXA_2_).

**Table 2:** Natural compounds in cardioprotection: evidence from models of cardiotoxicity and Cardiomyopathy. ↑: increase; ↓: decrease.

Source	Bioactive Compounds	Experimental Model	Treatment	Effects	Ref.
*Epimedii Herba* (Berberidaceae)	Icariin	In vitro study (H9C2 cell line)	Pre-treatment with Icariin (3 h): 1 µM, 5 µM, 10 µM, 20 µM Treatment with Doxo (24 h): 1 µM	↑ Cellular vitality; ↓ ROS; ↓ CAV-1; PDE5a Inhibition; ↓ Beclin-1; LC3.	[53]
*Ferula communis* L. (Apiaceae)	Ferutinin	In vitro study (H9C2 cell line)	Pre-treatment with Ferutinin (3 h): 0.15 μM 0.25 μM,1 μM, 2.5 μM, 5 μM, 7.5 μM, 10 μM 12.5 μM, 15 μM, 20 μM. Treatment with Doxo (24 h): 0.3 μM, 0.5 μM, 1 μM, 3 μM.	↑ Cellular viability; ↓ ROS; Protection against Doxo-induced cardiotoxicity; Restoration of S phase, G2/M.	[55]
*Citrus Bergamia* Risso et poiteau (Rutacee)	Bergamot polyphenolic fraction (BPF): brutieridin, melitidin, naringin, neohesperidin	In vitro study (H9C2 cell line)	Pre-treatment with BPF: 5 µg/mL, 10 µg/mL, 25 µg/mL Treatment with Doxo (24 h): 1 µM	↑ Cellular viability; ↓ ROS; Anti-apoptotic effect; Mitochondrial protection;	[56]
*Cynara cardunculus* L. (Asteraceae)	Caffeoylquinic acids, flavonoids, Cynaropicrin.	In vitro study (H9C2 cell line)	Pre-treatment with Cynara extract: 1 µg/mL, 5 µg/mL, 10 µg/mL Treatment with Doxo (24 h): 1 µM	↑ Cellular viability; ↓ ROS; Anti-apoptotic effect.	[57]
*Vitis vinifera* L. (Magnoliopsida)	Resveratrol	In vivo study (rats with diabetic cardiomyopathy induced by high-fat diet + STZ)	Resveratrol 50 mg/kg/day orally (16 weeks)	↑ Cardiac function; ↓ Mitochondrial dysfunction; ↓ Hypertrophy; Antioxidant effects.	[58]
*Allium cepa* L. (common onion) apples, berries, tea	Quercetin	In vivo study (streptozotocin-induced diabetic mouse model with high-fat diet)	Quercetin, 100 mg/kg/day, oral after streptozotocin injection	↑ Cardiac contractile function; ↓ Myocardial inflammation; ↓ Cardiac fibrosis; Modulation of glycerophospholipid metabolism.	[59]
*Curcuma Longa* L. (Zingiberaceae)	Curcumin	In vivo study (diabetic rats) + in vitro study cardiomyocyte model	In vivo: Curcumin 200 mg/kg/day orally in diabetic rats In vitro: Curcumin 14 μM applied to high-glucose–treated cardiomyocytes	↑ Cardiac function and viability; ↓ Cardiomyocyte pyroptosis; ↓ ROS; AKT/Nrf2/ARE Activation;	[60]
*Haematococcus pluvialis*	Astaxanthin	In vivo study (adult male Wistar rats with high-fat/high-fructose diet + STZ-induced diabetic cardiomyopathy)	Astaxanthin 100 mg/kg/day orally (4 weeks)	↑ Cardiac function and ECG parameters; ↓ MDA, ↑ GSH, ↑SOD; ↓ NOX4; ↑ Nrf2/ARE; ↓ AP-1.	[61]
*Cyclocarya paliurus* (Juglandaceae)	Quercetin, kaempferolisoquercetin, caffeic acid	In vivo study (db/db diabetic mice model of diabetic cardiomyopathy)	Daily oral gavage of Cyclocarya paliurus ethanol leaf extract (10 weeks): 20 mg/kg/day (low dose) 40 mg/kg/day (medium dose) 80 mg/kg/day (high dose)	↓ Blood glucose; ↓ TG, ↓ TC, ↓ MDA; ↑ SOD, ↑ GSH-Px, ↑ CAT, ↓ TNF-α, ↓ IL-1β, ↓ IL-6, ↓ Myocardial fibrosis.	[62]
*Capparis spinosa* L. (Capparaceae)	Polyphenols (enriched fraction CSN-50%; includes flavonoids and phenolic compounds)	In vitro study (cardiomyocytes) In vivo study (diabetic mice model of diabetic cardiomyopathy)	Treatment with polyphenol-enriched fraction CSN-50% obtained from *Capparis spinosa* via ultrasound-assisted ethanol extraction and purification (in vitro and in vivo administration.	↑ Antioxidant activity; ↓ ROS; ↓ Cardiomyocyte apoptosis; ↓ Blood glucose; ↓ Cardiac fibrosis; Potential involvement of Nrf2 and PI3K/Akt pathways.	[63]
*Phoenix dactylifera* L. (Arecaceae)	Quercetin, kaempferol, luteolin, apigenin, gallic acid, caffeic acid	In vivo study (male Wistar rats; STZ-induced diabetic cardiomyopathy model)	Treatment with Phoenix dactylifera methanolic extract: 5 mg/kg/day (oral administration) (25 days)	↓ blood glucose; ↓ cholesterol; ↑ insulin signaling; ↓ TGF-β; ↑ Myocardial structure and function.	[64]
*Camellia sinensis* L. (Theaceae)	Epigallocatechin gallate (EGCG), catechins	In vivo study (male Sprague–Dawley rats; 5/6 nephrectomy-induced cardiac hypertrophy model); In vitro study (isolated adult rat cardiomyocytes)	In vivo: green tea extract: 0.1% and 0.25% concentrations (4 weeks) In vitro: cardiomyocytes treated with green tea extract in presence of ouabain/marinobufagenin (MBG).	↓ left ventricular ↓ hypertrophy; ↓ hypertension; ↓ ROS; Na^+^/K^+^-ATPase activity Preservation; Inhibition of cardiomyocyte hypertrophic response.	[65]
*Zingiber officinale* (Zingiberaceae)	Gingerols, Shogaols.	In vitro study (cardiomyocytes hypertrophy model); In vivo study (mouse model of pressure overload-induced heart failure)	In vitro: compound A (1 µM) Stimulated with phenylephrine (cardiomyocytes) or TGF-β (fibroblasts) In vivo: C57BL/6J mice subjected to transverse aortic constriction Treated with compound A 1 mg/kg/day (oral administration) (8 weeks).	↓ Cardiomyocyte hypertrophy; ↓ Cardiac fibrosis; Prevention of progression to heart failure.	[66]
*Andrographis paniculata* (Acanthaceae)	Andrographolide, γ-sitosterol, stigmasterol, cortolone, and other diterpenoids/flavonoids	In vitro study (H9c2 rat cardiomyoblast cell line; Angiotensin II-induced cardiac hypertrophy model)	Pre-treatment with Andrographis paniculata ethanolic extract (APE): 9 µg/mL Hypertrophy induction with Angiotensin II (1 µM).	↓ Cardiomyocyte hypertrophy (↓ cell size, ↓ BNP, ↓ AT1R expression) ↓ ROS ↑ Antioxidant response (↑ Nrf2, ↑ SOD1 expression) ↓ Inflammation (↓ NF-κB, ↓ NLRP3, ↓ IL-1β) Modulation of Nrf2/NF-κB/NLRP3 signalling pathway	[67]
*Allium sativum* L. (Amaryllidaceae)	Allicin, alliin, and other organosulfur compounds	In vitro study (rat cardiomyocytes; norepinephrine-induced hypertrophy model)	Pre-treatment with garlic extracts: 4 µL, 10 µL, 20 µL per 4 mL culture medium (30 min) pre-treatment Hypertrophy induction with norepinephrine (0.25 µM) (24 h)	↓ Cardiomyocyte hypertrophy (↓ cell surface area, ↓ protein synthesis); Anti-hypertrophic effect mediated by NO and H_2_S signalling pathways.	[68]
*Nelumbo nucifera* (Nelumbonaceae)	lotus bee pollen extract; e.g., quercetin derivatives, kaempferol derivatives	In vitro study (H9c2 rat cardiomyoblast cell line; isoproterenol-induced hypertrophy model)	Pre-treatment with lotus bee pollen extract (LBPE): 50, 100, 200 µg/mL Hypertrophy induction with isoproterenol (10 µM) (24 h).	↓ Cardiomyocyte hypertrophy (↓ cell surface area, ↓ protein synthesis); ↓ ROS; ↓ Apoptosis; Inhibition of JAK2/STAT3 signalling pathway.	[70]
*Olea europaea* L. (Oleaceae)	Oleuropeinhydroxytyrosol, tyrosol and other phenolic compounds	In vivo study (male rats; streptozotocin (STZ)-induced diabetic cardiomyopathy model)	Treatment with olive leaf extract (OLE): 100 mg/kg/day 200 mg/kg/day 400 mg/kg/day (6 weeks); Metformin 300 mg/kg/day Valsartan 30 mg/kg/day	↓ HW/BW ratio; ↓ ANP, BNP, β-MHC expression; ↓ Cardiac fibrosis (↓ TGF-β1, TGF-β3, collagen, α-SMA); ↓ Blood glucose levels; ↓ Activation of AT1 receptor signalling.	[71]
*Achyranthes bidentata* (Amaranthaceae) *Saururus chinensis* (Saururaceae)	Quercetin, inophyllum E	In silico, in vitro and in vivo study	In vitro: treatment with quercetin in angiotensin II-induced cardiomyocyte model In vivo: validation of target gene expression.	Quercetin showed protective effects against cardiomyocyte stress; Potential regulation of pathways involved in hypertrophic cardiomyopathy and hypertension Multi-target mechanism based on network pharmacology approach.	[72]

Ak strain transforming (AKT); nuclear factor erythroid 2-related factor 2 (Nrf2); Antioxidant Response Element (ARE); atrial natriuretic peptide (ANP); Activator Protein-1 (AP-1); Andrographis paniculata ethanolic extract (APE); angiotensin 1(AT1); angiotensin receptor 1 (AT1R); B-type Natriuretic Peptide (BNP); Bergamot Poliphenolic Fraction (BPF); catalase (CAT); Caveolin-1 (CAV-1); doxorubicin (DOX); electrocardiographic (ECG); Epigallocatechin gallate (EGCG); glutathione (GSH); glutathione peroxidase (GSH-Px); hydrogen sulfide (H_2_S); Heart Weight to Body Weight ratio (HW/BW); Interleukin-1β (IL-1β); Interleukin-6 (IL-6); Janus Kinase 2/Signal Transducer and Activator of Transcription 3 (JAK2/STAT3); lotus bee pollen extract (LBPE); Protein 1 Light Chain 3 (LC3); marinobufagenin (MBG); Malondialdehyde (MDA); Sodium-Potassium Adenosine Triphosphatase (Na^+^/K^+^-ATPase); nuclear factor kappa-light-chain-enhancer of activated B cells (NF-kB); NLR family pyrin domain containing 3 (NLRP3); nitic oxide (NO); NADPH oxidase 4 (NOX4); olive leaf extract (OLE); Phosphodiesterase 5A (PDE5a); Phosphoinositide 3-kinase/ak strain transforming (PI3K/Akt); reactive oxygen species (ROS); superoxide dismutase (SOD); Streptozotocin (STZ); total cholesterol (TC); total triglycerides (TG); transforming growth factor-beta (TGF-β); transforming growth factor-beta 1 (TGF-β1); transforming growth factor-beta 3 (TGF-β3); Tumor Necrosis Factor-alpha (TNF-α); Alpha-Smooth Muscle Actin (α-SMA); β-myosin heavy chain (β-MHC).

## Data Availability

No new data were created or analyzed in this study. Data sharing is not applicable to this article.

## Abbreviations

8-iso-PGF2α	8-iso-prostaglandin F2α
AA	arachidonic acid
AHA	American Heart Association
AKBA	acetyl-keto-β-boswellic acid
Akt	ak strain transforming
ALX/FPR2	lipoxin A4 receptor/formyl peptide receptor 2
ANGII	angiotensin II
ANP	atrial natriuretic peptide
AP 1	Activator Protein-1
APE	Andrographis paniculata ethanolic extract
ARE	Antioxidant Response Element
ARVC	arrhythmogenic right ventricular cardiomyopathy
ASTA	Astaxanthin
AT1	angiotensin 1
AT1R	angiotensin receptor 1
ATP	adenosine triphosphate
Bcl 2/Bax	B-cell lymphoma/leukemia-2/Bcl-2-associated X protein
BLT_1_	leukotriene B4 receptor 1
BLT_2_	leukotriene B4 receptor 2
BNP	B-type Natriuretic Peptide
BPF	bergamot polyphenolic fraction
CAD	coronary artery disease
cAMP	cyclic adenosine monophosphate
CAT	catalase
CAV-1	Caveolin-1
CHD	congenital heart disease
ChemR23	chemerin receptor 23
CoQ10	Coenzyme Q10
COX	cyclooxygenases
cPLA2	cytosolic phospholipase A2
CRTH2	chemoattractant receptor expressed on type 2 helper T cells
CYP450	cytochrome P450
CysLT	cysteinyl leukotrienes
CysLT_1_	cysteinyl leukotrienes-1
CysLT_2_	cysteinyl leukotrienes-2
DCM	diabetic cardiomyopathy
DGLA	dihomo-γ-linolenic acid
DHA	docosahexaenoic acid
DIC	DOX-induced cardiotoxicity
DM	diabetes mellitus
DOX	doxorubicin
DP1	D-prostanoid receptor 1
DP2	D-prostanoid receptor 2
DQP	DanQi Pill
EETs	epoxyeicosatrienoic acids
EF	ejection fraction
EGCG	epigallocatechin gallate
EP1	prostaglandin E2 receptor 1
EP2	Prostaglandin E receptor 2
EP3	prostaglandin E2 receptor 3
EP4	prostaglandin E2 receptor 4
EP4 KO	prostaglandin E receptor 4 knockout
EPA	eicosapentaenoic acid
ER	endoplasmic reticulum
ETC	electron transport chain
FP	prostaglandin F
GCL	glutamate cysteine ligase
GPCR32	G protein-coupled receptor 32
GPCRs	G protein-coupled receptors
GSH	glutathione
GSH-Px	glutathione peroxidase
Gα12/13	G protein alpha 12 and alpha 13 subunits
Gαi/o	Inhibitory/Other G protein alpha subunits
Gαq/11	G protein alpha q and alpha 11 subunits
Gαs	stimulatory G protein alpha subunit
H2S	hydrogen sulfide
H9C2	rat embryonic cardiomyoblast cell line
HCM	hypertrophic cardiomyopathy
HDAC5	histone deacetylase 5
HDAC-MEF2	histone deacetylase-myocyte enhancer factor 2
HETEs	hydroxyeicosatetraenoic acids
HF	Heart Failure
HO-1	heme oxygenase-1
HPETE	hydroperoxyeicosatetraenoic acid
HpFA	hydroperoxy fatty acid
HW/BW	Heart Weight to Body Weight ratio
ICA	Icariin
IP	prostaglandin I_2_
IPKO	prostaglandin I2 receptor knockout
iPLA_2_	calcium-independent phospholipases A_2_
JAK2/STAT3	Janus Kinase 2/Signal Transducer and Activator of Transcription 3
LBPE	lotus bee pollen extract
LC3	Protein 1 Light Chain 3
LOX	lipoxygenases
LPS	lipopolysaccharide
LT	leukotrienes
LTA_4_	leukotriene A4
LTB_4_	leukotriene B4
LTB4R	leukotriene B4 receptor
LTC_4_	leukotriene C4
LTD_4_	leukotriene D4
LTE_4_	leukotriene E4
LXA_4_	lipoxin A_4_
LXB_4_	lipoxin B_4_
LXRα	liver X receptor α
MAPK	mitogen-activated protein kinase
MBG	marinobufagenin
MCP-5	monocyte chemotactic protein-5
MDA	malondialdehyde
MEF2	myocyte enhancer factor 2
MMP9	Matrix Metalloproteinase-9
mRNA	messenger ribonucleic acid
Myh7	brain natriuretic peptide and atrial natriuretic peptide
Na^+^/K^+^-ATPase	Sodium-Potassium Adenosine Triphosphatase
NDLVC	non-dilated ventricular cardiomyopathy
NF-kB	nuclear factor kappa-light-chain-enhancer of activated B cells
NLRP3	NLR family pyrin domain containing 3
NO	nitic oxide
NOX4	NADPH oxidase 4
Nrf2	nuclear factor erythroid 2-related factor 2
NSAIDs	non-steroidal anti-inflammatory drugs
OXE1	oxoeicosanoid receptor 1
P/T	prostaglandin I2/thromboxane A2
PAK2	p21-activated kinase 2
p-Akt	phosphorylated Ak strain transforming
PDE5a	Phosphodiesterase 5A
PG	prostaglandins
PGC-1α	Peroxisome proliferator-activated receptor gamma coactivator 1-alpha
PGD_2_	prostaglandin D2
PGE_2_	prostaglandin E2
PGF2α	prostaglandin F2α
PGG_2_	prostaglandin G_2_
PGH_2_	prostaglandin H_2_
PGI_2_	prostaglandin I2
PGI3	prostaglandin I3
PI3K/Akt	Phosphoinositide 3-kinase/ak strain transforming
PKD	protein kinase D
PLA_2_	phospholipases A_2_
PLN	phospholamban
PNS	panax notoginseng
PUFA	polyunsaturated fatty acids
Rac1	ras-related C3 botulinum toxin substrate 1
RCM	restrictive cardiomyopathy
ROS	reactive oxygen species
SCD	sudden cardiac death
SERCA2a	sarcoplasmic and endoplasmic reticulum calcium-ATPase 2a
SIRT1	Sirtuin 1
SOD	superoxide dismutase
sPLA_2_	secreted phospholipases A_2_
SR	sarcoplasmic reticulum
STAT3	Signal Transducer and Activator of Transcription 3
STZ	Streptozotocin
T2D	type 2 diabetes mellitus
TC	total cholesterol
TCM	traditional chinese medicine
TG	total triglycerides
TGF-β	transforming growth factor-beta
TGF-β1	transforming growth factor-beta 1
TGF-β3	transforming growth factor-beta 3
Tiam1	T-cell lymphoma invasion and metastasis 1
TNF-α	Tumor Necrosis Factor-alpha
TP	thromboxane prostanoid
TX	thromboxanes
TXA2	thromboxane A2
TXB_2_	thromboxane B2
WHO	world health organization
α-SMA	Alpha-Smooth Muscle Actin
β-MHC	β-myosin heavy chain
